# SHARP SEATTLE: A COMMUNITY COLLABORATION TO CREATE BLACK HISTORY WALKING ROUTES AND ENGAGE WALKERS

**DOI:** 10.1093/geroni/igae098.1267

**Published:** 2024-12-31

**Authors:** Anthony Cryer, Stephanie Johnson-Toliver, Dian Ferguson, Juell Towns

**Affiliations:** Central Area Senior Center, Seattle, Washington, United States; Black Heritage Society of Washington State, Seattle, Washington, United States; Central Area Senior Center, Seattle, Washington, United States; University of Washington, Seattle, Washington, United States

## Abstract

The Sharing History through Active Reminiscence and Photo-Imagery (SHARP) program honors Black health and history through creating culturally celebratory walking routes. Nine community and academic partners implemented SHARP Seattle in the gentrifying Central District. Key to success was partners with established community relationships and expertise aligned with the SHARP approach, including in walking programs, archival images, local Black history, cognitive health, and the needs, concerns, and preferences of older Black adults regarding programming. Study activities were based at and coordinated by a senior center serving primarily African Americans. Student interns led informant focus groups with 10 older Black adults to gather themes and topics for inclusion in twelve 1-mile walking routes. Interns worked with an archivist on selecting local historical images to illustrate community-identified walk themes and solicit stimulating conversational reminiscence among walkers. Themed routes with images were programmed into the SHARP walking application, tested, and revised. After a kick-off celebration, thirty-one older Black adults, grouped into triads, engaged 3 times a week for 4 weeks to walk the 12 routes. In this talk, community partners discuss their experience, including learning the research process, exerting their preference for an adapted, more relaxed study design for less burden and greater inclusion of people with cognitive impairment, creating routes with different generations whose local geo-historical knowledge differed greatly, strategies for curating community buy-in, gentrification and displacement-related recruitment challenges, and learning from participant feedback. Finally, self-reflections explore the meaningfulness of walking and social reminiscence amidst aging and gentrification.

